# Lymphoepithelioma-Like Carcinoma of the Lung: An Unusual Case and Literature Review

**DOI:** 10.1155/2013/143405

**Published:** 2013-10-30

**Authors:** Yuan-Chun Huang, Ching Hsueh, Shang-Yun Ho, Chiung-Ying Liao

**Affiliations:** ^1^Department of Medical Imaging, Changhua Christian Hospital, No. 135 Nanxiao Street, Changhua 500, Taiwan; ^2^Department of Radiology, Changhua Christian Hospital, Erlin Branch, No. 558, Section 1, Dacheng Road, Erlin Township, Changhua 526, Taiwan

## Abstract

We described a case of lymphoepithelioma-like carcinoma (LELC) of the lung of a 65-year-old man with initial symptoms of intermittent chest pain and mild shortness of breath for 2 weeks. A right-lung mass was noted on chest computed tomography (CT) scan and was proved histopathologically as LELC of lung after video-assisted thorascopic lobectomy. He was successfully treated with lobectomy with postoperative adjuvant chemotherapy and is alive without signs of recurrence for 36 months after the diagnosis. It is important for clinicians, pathologists, and radiologists to understand the clinical, pathological, and radiological presentations of this neoplasm to avoid improper clinical decision making and misdiagnosis.

## 1. Introduction

LELC of the lung was first reported in 1987 [1]. Primary LELC of the lung is a rare entity that has recently been included as a subtype of variants of large cell carcinoma in the World Health Organization's histologic classification of lung tumors [2]. Being a rare entity and mostly seen in Asians, few cases have been described previously [3]. The behavior of LELC of the lung is reported to be highly variable [4]. LELC has been reported in pharyngeal and foregut derivatives including the oral cavity, salivary glands, thymus, lungs, and stomach [5]. The association with Epstein-Barr virus (EBV) is variable [6]. Primary LELC of the lung is rare. The literature of LELC of the lung involves just more than 150 cases until 2006 [3]. In majority, those patients are Orientals, with nearly two-thirds arising from Taiwan, Southern China, and Hong Kong [3].

We present an unusual case with a pulmonary mass on CT scan of the thorax which was subsequently proved as a LELC of the lung and a brief review of the relevant literature.

## 2. Case Report

**Figure 1:**
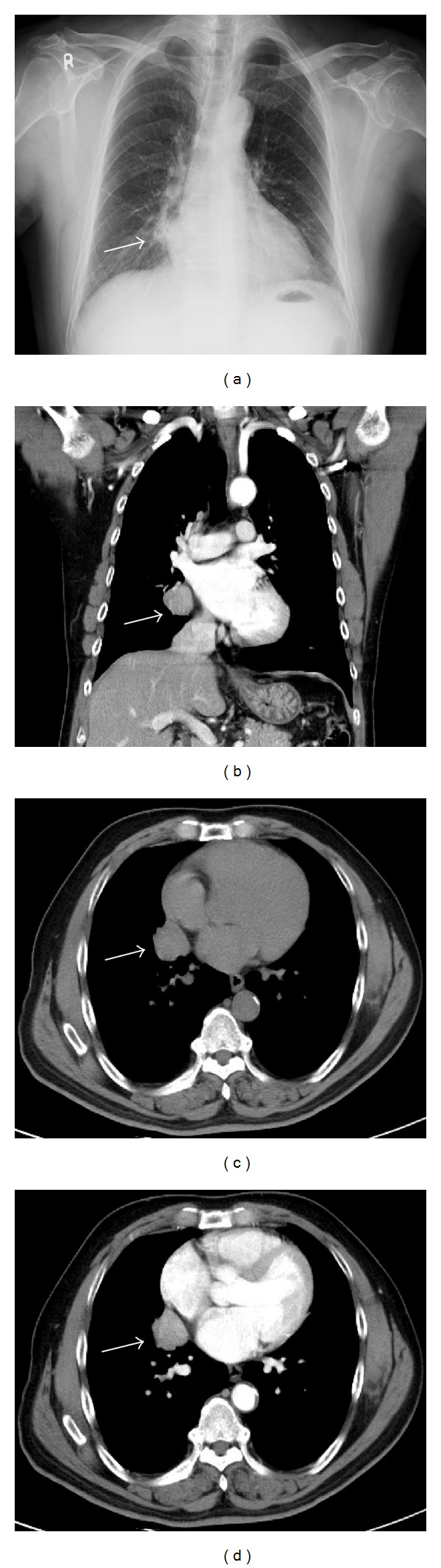
(a) Chest X-ray showed a mass lesion in the right paramediastinal region (arrow); (c) Noncontrast-enhanced CT scan: an isodensity lobulated mass lesion in the right middle lobe of lung; ((b) and (d)) Chest CT scan showed a 30 × 29 mm heterogenously enhanced mass lesion with well-defined lobulated margin in the right middle lobe of lung, abutting the mediastinum.

The patient is a 65-year-old Taiwanese man, a businessman with initial symptoms of intermittent chest pain with mild shortness of breath for two weeks. Chest X-ray showed a mass lesion in the right lower lung field. Chest CT scan showed a 30 × 29 mm heterogeneously enhanced mass lesion with well-defined margin and lobulated contour in the right middle lobe of lung, abutting the mediastinum (Figure 1). Bronchoscopy showed no endobronchial lesion. He received video-assisted thorascopic lobectomy of right middle lobe of lung and mediastinal lymph nodes dissection.

**Figure 2:**
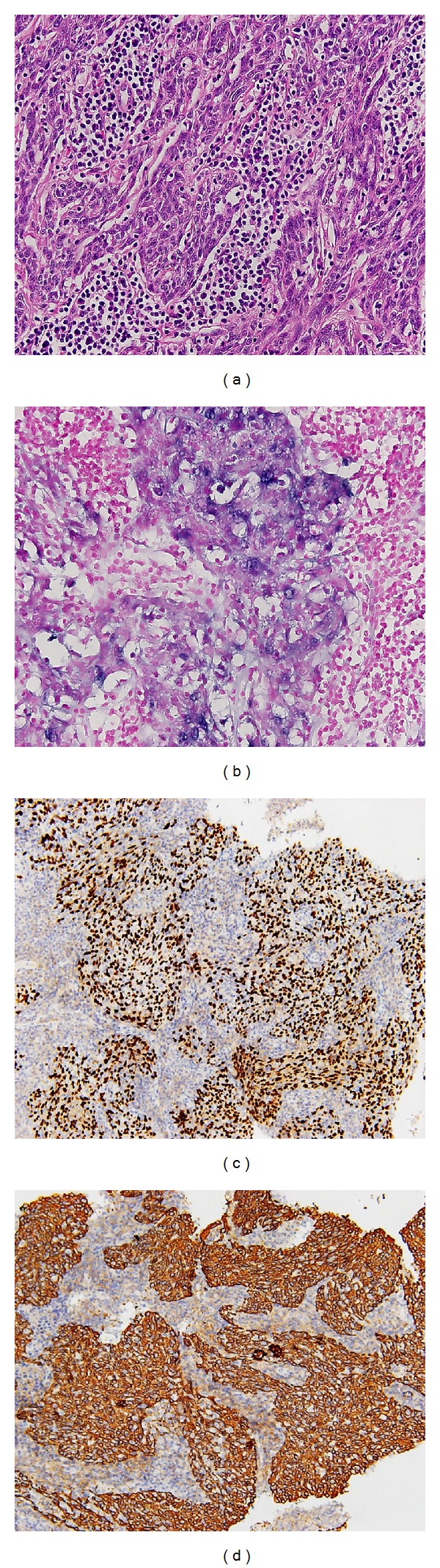
(a) Hematoxylin- and Eosin-stained cell block section shows non-small cell carcinoma consisting of syncytial tumor cells with focal necrosis and lymphocytic infiltrate in the background (original magnification, ×400); (b) The immunohistochemical study: EBER(+) (original magnification, ×400); (c) The immunohistochemical study: P63(+) (original magnification, ×200); (d) The immunohistochemical: CK(+) (original magnification, ×200).

The pathology, immunohistochemical staining, and in situ hybridization results confirmed LELC of lung. Microscopically, the tumor cells are surrounded by abundant lymphoplasmacytic cells in the stroma. The tumor cells show indistinct cell borders with prominent nucleoli and are closely admixed with infiltrating inflammatory cells. Using in situ hybridization with exhibition of abundant EBV-encoded small nuclear RNA (EBER) in the majority of tumor cells is done, which has become a standard test to display tumor-specific association of EBV. Immunohistochemical staining was positive for cytokeratin (CK), a marker which was almost always positive in LELC of lung [7]. Immunohistochemical staining for P63 was positive. P63 protein as homologue of the p53 protein, being a powerful marker for squamous differentiation, was expressed, which excluded a glandular or neuroendocrine differentiation (Figure 2).

Head and neck CT scan and nasopharyngeal fiberscopy were performed and no obvious tumor was found. The patient's postoperative course was uncomplicated, and he was discharged 7 days after operation. Due to advanced stage with parietal pleura invasion and presence of subcarinal lymph node metastasis, postoperative adjuvant chemotherapy was performed on schedule.

## 3. Discussion

In the literature, most imaging characters of advanced primary pulmonary LELC have been reported in several small clinicopathologic studies en passant [8–10]. Ooi et al. brought out a comparison of CT features between advanced-stage patients (stages III and IV) with LELC of lung and non-small cell lung carcinoma [11]. Those authors stated that LELC of lung was inclinable to demonstrate the following features: central location, large size, smooth margin, vascular encasement, and peribronchovascular nodal spread. Ooi and colleagues stated that if large pulmonary lesions were closely associated with the mediastinum, especially during the occurrence of vascular encasement and peribronchovascular nodal spread, the diagnosis of primary LELC of lung is more likely than non-LELC neoplasms. Notwithstanding, these features observed by Ooi et al. may be present in patients with bronchogenic carcinoma. Moreover, Chan et al. and Han et al. studied late-stage lesions, and therefore their findings could not be applied completely to earlier-stage patients [9, 10].

The results of Chan et al. [9], Han et al. [10], and Hoxworth et al. [12] suggested that primary LELC of lung most often manifests itself as a peripheral poorly marginated nodule, smaller than 3.5 cm in size, and usually is not associated with lymphadenopathy. However, Ooi et al. declare that primary LELC of lung usually presents as a large pulmonary mass in the central third of the lung with circumscribed borders and associated with lymphadenopathy. The CT scan findings of our case in this report were compatible with the latter descriptions.

Hoxworth et al. [12] first described the MRI features of LELC of the lung. MRI findings of primary pulmonary LELC include intense enhancement with iso- to hypointensity on T1-weighted sequences and iso- to hyperintensity on T2-weighted sequences. Unfortunately, these MRI signal features are nonspecific. As a consequence, the role of MRI in evaluating LELC will be limited as preoperative planning and staging tool with detection of adjacent structures invasion.

In Oriental populations, there is a close relationship between EBV infection and pulmonary LELC. EBV infection may have an essential role in the tumorigenesis of pulmonary LELC [13]. The presence of EBV in LELC has been demonstrated by polymerase chain reaction for EBV DNA, in situ hybridization for EBV DNA and RNA, and immunohistochemistry for EBV-associated proteins [14, 15]. However, it is suggested that there is no association between EBV and LELC in the Western population [16]. Furthermore, a detail expression profile of EBV viral proteins in pulmonary LELC has not been reported.

LELC is pathologically a distinct entity which was classified as a type of non-small cell lung cancer [9]. In histology, it is indistinguishable between primary LELC of the lung and the prototypical LELC occurring in the nasopharynx [9]. Consequently, a nasopharyngeal origin needs to be excluded in all cases. A thorough evaluation of other primary sites such as the nasopharynx should be carried out. The incidence of metastasis to local lymph nodes is 25%; although hematogenous metastasis occurs seldom, the skeletal system is the preferred site [17, 18].

Metastatic nasopharyngeal carcinoma and non-Hodgkin's lymphoma are two main differential diagnoses for LELC [8]. The latter commonly receives nonsurgical management. Incorrect diagnosis will lead to inaccurate staging and inappropriate management. Identification of primary pulmonary LELC will allow precise staging and proper patient management. In the subject of differentiation between lymphoma and LELC, immunohistochemical staining plays a significant role [9]. Neck magnetic resonance imaging or computed tomography scan cooperatively with endoscopic biopsy of the nasopharynx is essential to exclude primary nasopharyngeal carcinoma.

Surgery is the major curative method for stage I non-small cell carcinoma of the lung; patients with late stage non-small cell carcinoma of the lung such as stage II or higher are treated by combination therapy including postoperative radiotherapy, chemotherapy, or both.

LELC in the nasopharynx is radiosensitive, and increasingly it is being perceived as chemosensitive [19]. Ho et al. observed 7 patients with LELC of the lung for response to chemotherapy and found that 5 (71%) had a partial response and 2 (29%) had progressive disease [20]. Evidence about the role of radiotherapy and chemotherapy for LELC of the lung needs further study owing to the relatively small number of cases. However, chemotherapy and radiotherapy have been employed with some success [10, 21].

From limited data available, the behavior of LELC of lung is highly variable nevertheless aggressive malignancy is reported in the minority of cases [5, 22].

Han et al. asserted that the overall survival rate is more favorable in LELC of the lung compared with non-LELC type of non-small cell lung carcinoma; furthermore, it was found that tumor recurrence and necrosis were poor prognostic factors for survival [10]. However, other factors inherent to the nature of the carcinoma may play a part in its relatively good prognosis. The presence of abundant CD8-positive cytotoxic T lymphocytes adjacent to LELC cells and the underexpression of p53 and c-erb B-2 oncoproteins in tumor cells have been postulated to account for the better prognosis in LELC of the lung [21].

## 4. Conclusion

Conclusively, the CT and MRI image findings of primary pulmonary LELC are similar to those of bronchogenic carcinomas in the majority of cases. LELC of lung may be mistaken histopathologically for metastatic nasopharyngeal carcinoma or lymphoma, resulting in improper patient management. LELC should be considered in the differential diagnosis of primary lung tumors, particularly when an extensive lymphocytic infiltrate is observed. Clinicians, pathologists, and radiologists may encounter primary pulmonary LELC on imaging or at biopsy procedure; consequently familiarity with this distinctive entity is required.
